# c-KIT and c-KIT ligand (SCF) in synovial sarcoma (SS): an mRNA expression analysis in 23 cases

**DOI:** 10.1054/bjoc.2001.1935

**Published:** 2001-08

**Authors:** E Tamborini, D Papini, A Mezzelani, C Riva, A Azzarelli, G Sozzi, M A Pierotti, S Pilotti

**Affiliations:** Department of Pathology, Istituto Nazionale per lo Studio e la Cura dei Tumori, Milano, 20133, Italy; Department of Musculo-Skeletal Surgery, Istituto Nazionale per lo Studio e la Cura dei Tumori, Milano, 20133, Italy; Department of Experimental Oncology, Istituto Nazionale per lo Studio e la Cura dei Tumori, Milano, 20133, Italy

**Keywords:** synovial sarcoma, RT-PCR, c-KIT/SCF, anti-apoptotic gene

## Abstract

In a previous immunophenotypic molecular-based analysis it was shown that bcl2 over-expression characterizes the SS gene profile in addition to the non-random translocations. Here we show that the over-expression of an additional potentially antiapoptotic gene, the c-KIT gene, is associated with this tumour. Interestingly, whereas bcl2 over-expression appears to be restricted to the spindle cell tumoral component, c-kit mainly involves the epithelial component of biphasic SS. Twenty-three primary and metastatic samples from 21 patients were analysed by immunophenotyping (23/23), immunoprecipitations and Western blotting (3/23), and RT-PCR (23/23). Ten cases were biphasic and 13 monophasic in sub-type. Twelve, 10 and 1 case carried the SYT-SSX1, SYT-SSX2 and SYT-SSX4 fusion transcript, respectively. Co-presence of both c-Kit and SCF mRNA was observed in almost all cases (20/23), suggesting the occurrence of an autocrine loop. Immunophenotyping, confirmed by biochemical analyses, showed a modulation of c-Kit expression which was faint in the spindle and strong in the epithelial component, respectively. The study was complemented by c-Met/HGF receptor/ligand expression and c-Met protein analysis with results superimposable to those already reported. Since in each tumour, epithelial and spindle cell components harbour the same type of translocation t(X;18) the present findings suggest a shifting of the anti-apoptotic role from BCL2 to c-KIT gene during the transition from the uncommitted spindle to the differentiated epithelial cells. © 2001 Cancer Research Campaign http://www.bjcancer.com


*British Journal of Cancer* (2001) **85**(3), 405–411
© 2001 Cancer Research Campaign
doi: 10.1054/ bjoc.2001.1935, available online at http://www.idealibrary.com on IDEAL®

### c-KIT and c-KIT ligand (SCF) in synovial sarcoma (SS): an mRNA expression analysis in 23 cases

**E Tamborini[1], D Papini[1], A Mezzelani[1], C Riva[1], A Azzarelli[2], G Sozzi[3], MA Pierotti[3] and S Pilotti[1]**

[1]Department of Pathology; [2]Department of Musculo-Skeletal Surgery, and [3]Department of Experimental Oncology, Istituto Nazionale per lo Studio e la Cura dei Tumori, 20133 Milano, Italy

**Summary** In a previous immunophenotypic molecular-based analysis it was shown that bcl2 over-expression characterizes the SS gene profile in addition to the non-random translocations. Here we show that the over-expression of an additional potentially antiapoptotic gene, the c-KIT gene, is associated with this tumour. Interestingly, whereas bcl2 over-expression appears to be restricted to the spindle cell tumoral component, c-kit mainly involves the epithelial component of biphasic SS. Twenty-three primary and metastatic samples from 21 patients were analysed by immunophenotyping (23/23), immunoprecipitations and Western blotting (3/23), and RT-PCR (23/23). Ten cases were biphasic and 13 monophasic in sub-type. Twelve, 10 and 1 case carried the SYT-SSX1, SYT-SSX2 and SYT-SSX4 fusion transcript, respectively. Co-presence of both c-Kit and SCF mRNA was observed in almost all cases (20/23), suggesting the occurrence of an autocrine loop. Immunophenotyping, confirmed by biochemical analyses, showed a modulation of c-Kit expression which was faint in the spindle and strong in the epithelial component, respectively. The study was complemented by c-Met/HGF receptor/ligand expression and c-Met protein analysis with results superimposable to those already reported. Since in each tumour, epithelial and spindle cell components harbour the same type of translocation t(X;18) the present findings suggest a shifting of the anti-apoptotic role from BCL2 to c-KIT gene during the transition from the uncommitted spindle to the differentiated epithelial cells. © 2001 Cancer Research Campaign http://www.bjcancer.com

**Keywords**: synovial sarcoma; RT-PCR; c-KIT/SCF; anti-apoptotic gene

Recently, deregulation of the expression of proto-oncogenes belonging to the growth factor receptor family with intrinsic thyrosine kinase activity, has been reported in SSs. Co-expression of c-MET and HGF/SF and expression of IgF-1R has been shown in SS surgical specimens, the former by immunocytochemistry (Fukuda et al, 1998; Motoi et al, 1998; Kuhnen et al, 1998; Oda et al, 2000), by Western blotting (Wb) and by RT-PCR (Fukuda et al, 1998; Oda et al, 2000) and the second by Wb and RT-PCR (Xie et al, 1999). In accordance with the epithelial differentiation driving role of c-MET in the c-MET-HGF/SF system, c-MET immunoreactivity has been mainly found in the epithelial component of tumours (Fukuda et al, 1998; Motoi et al, 1998; Kuhnen et al, 1998; Oda et al, 2000), even though, by virtue of its role in the promotion of progression, the co-expression of HGF and c-Met has been recently correlated with poor prognosis in SSs (Oda et al, 2000). Moreover, in keeping with the role played by IgF-IR in tumour cell growth, its expression has been found to be significantly associated with a high incidence of lung metastases (Xie et al, 1999).

The proto-oncogene c-Kit was identified as the cellular homologue of the oncogene c-Kit present in the genome of Hardy-Zuckerman 4-feline sarcoma virus that induces multicentric fibrosarcoma in domestic cats (Besmer et al, 1986). This proto-oncogene encodes a tyrosine kinase receptor which bears a region homologous with platelet-derived growth factor and colony stimulating factor 1 receptor (Yarden et al, 1987; Qui et al, 1987). The ligand for c-Kit product has been cloned and variously designated as stem cell factor (SCF) (Besmer et al, 1986), mast cell growth factor, kit ligand or steel factor (Williams et al, 1990; Copeland et al, 1990; Nocka et al, 1990). SCF-Kit interaction is essential for the development of melanocytes, germ cells and mast cells (Galli et al, 1993).

Beyond a c-Kit role in cellular proliferation and differentiation (Torihashi et al, 1999), c-Kit is involved in protecting cells from apoptosis (Ricotti et al, 1998) as demonstrated in neuroblastoma (Timeus et al, 1997).

Recently, a role of KIT mutations and c-Kit/SCF mRNA over-expression in the development of sarcomas has been suggested (Ricotti et al, 1998; Hirota et al, 1998). A constitutively activated mutated c-Kit protein has been demonstrated in 5 out of 49 gastrointestinal stromal tumours (GISTs) (Hirota et al, 1998) currently regarded to originate from the putative intestinal cell of Cajal on the basis of immunophenotypic (Hirota et al, 1998; Kindblom et al, 1998) and ultrastructural features (Kindblom et al, 1998). Subsequently, an identical somatic and germ-line c-Kit mutation was found in a patient and in her nephew both suffering from multiple GISTs (Nishida et al, 1998) and, more recently, in a mother and her natural daughter (Hirota et al, 2000). At present, after the seminal Hirota's report, there is no consensus on the frequency of c-Kit gene mutations in sporadic GISTs, ranging from 21 % (Moskaluk et al, 1999) to more than 50% (Lasota et al, 1999) although the expanded observations share the evidence of a high correlation between malignancy and c-Kit mutation. Nevertheless, virtually all GISTs with and without mutations, showed CD 117 immunoreactivity (Moskaluk et al, 1999, Lasota et al, 1999). Furthermore, the mRNA co-expression of c-Kit-SCF, possibly related to an autocrine loop able to protect the cells from

*Received 6 December 2000*
*Revised 25 April 2001*
*Accepted 30 April 2001*

*Correspondence to:* S Pilotti apoptosis, has been described in six cell lines derived from pPNET (Ricotti et al, 1998).

Here we report the expression of the c-Kit gene and its ligand SCF, complemented by the c-Met/HGF gene system expression and c-Kit/c-Met immunophenotype in a series of synovial sarcomas (SS) already known to carry the non-random t(X;18) and over-expressing the protein encoded by the master inhibitory gene bcl2 (Mancuso et al, 2000).

### MATERIALS AND METHODS

### Tumours and patients

A total of 23 synovial sarcomas from 20 patients were analysed in this study. There were 11 primary tumours, 2 local tumour relapses and 10 pulmonary metastases: 9 to the lung and 1 to the adrenal gland. Cases 19 and 20 had both primary tumour (19a, 20a) and metastasis (19b, 20b and 20c). Anatomical location of primary tumours is detailed in Table 1.

Morphologically, 10 cases were biphasic and 13 monophasic in sub-type. A total of 12 cases showed the fusion transcript SYT-SSX1, 10 cases the SYT-SSX2 transcript and 1 the SYT-SSX4 one. All the SS cases in the study but 4 (case nos 7, 9, 20a and 20c) have been previously reported (Mancuso et al, 2000).

### Cell lines

The human megakaryoblastic leukaemia M07e cell line was kindly provided by Prof. L Pegoraro and was kept in culture as described by Brizzi et al, JBC 1994 (Brizzi et al, 1994). It was used as positive control for c-Kit and SCF expression.

FL-EBV cell line, derived from the fetal liver of a health donor immortalized with Epstein–Barr Virus, kindly provided by Dr Domenico Delia, was used as positive control for c-Met and HGF genes expression and was maintained in culture with 15% FCS RPMI in standard conditions.

### RNA extraction and reverse transcriptase polymerase reaction (RT-PCR)

Total RNA was extracted using the RNAzol method (Gibco BRL, Life Technology) from snap-frozen tumour tissue samples stored at –80˚C. 1 μg of RNA was reverse-transcribed into cDNA using oligo (dT) primers and reverse transcriptase (Superscript II Gibco BRL, Paisley, UK) according to the manufacturer's recommendations. The integrity of cDNA was detected by the amplification of the housekeeping β-actin gene (Adams et al, 1995). One μl of cDNA was used as template for each PCR reaction.

### SYT-SSX PCR

The detection of the putative SYT-SSX1 and SYT-SSX2 was carried out with the following primers:

SYT 5′-CAACAGCAAGATGCATACCA-3′
SSX1 5′-GGTGCAGTTGTTTCCCATCG-3′
SSX2 5′-GGCACAGCTCTTTCCCATCA-3′

PCR conditions were: 35 cycles of denaturation at 94˚C for 30 s, annealing at 58˚C for 1 min and elongation at 72˚C for 1 min (Kawai et al, 1998).

The detection of fusion transcript SYT-SSX4 was performed by a nested PCR with the following primer pairs:

**Table 1** Summary of the data

| Patient No. | Sample analysed | Age / sex site | Morphologic sub-types | Fusion transcript SYT-SSX | c-Kit mRNA | SCF mRNA | c-Kit ICC | c-met mRNA | HGF mRNA | c-met ICC |
|---|---|---|---|---|---|---|---|---|---|---|
| 1 | P | 48/F thigh | Biphasic | 4 | + | +LS | + | + | + | + |
| 2 | P | 36/F leg | Monophasic | 2 | + | +LS | + | + | + | + |
| 3 | P | 18/M neck | Biphasic | 1 | + | +LS | + | + | + | + |
| 4 | P | 46/F thigh | Monophasic | 1 | + | +LS | + | + | + | + |
| 5 | P | 48/M shoulder | Monophasic | 2 | + | +L | + | + | + | – |
| 6 | P | 39/F thigh | Monophasic | 2 | + | +LS | + | + | – | + |
| 7 | P | 12/M abdominal wall | Biphasic | 2 | + | +L | + | + | – | + |
| 8 | P | 50/F elbow | Biphasic | 2 | + | +L | + | + | + | – |
| 9 | P | 18/F sub-mandibular region | Biphasic | 1 | + | +LS | + | + | + | + |
| 10 | R | 52/F shoulder | Biphasic | 2 | + | +LS | + | + | + | + |
| 11 | R* | 63/F groin | Monophasic | 1 | + | +LS | + | + | + | + |
| 12 | AM* | 51/F thigh | Monophasic | 1 | + | +L | + | + | – | + |
| 13 | PM* | 30/M thigh | Monophasic | 2 | + | +LS | + | + | + | + |
| 14 | PM* | 54/F ankle | Monophasic | 1 | – | – | + | – | – | + |
| 15 | PM* | 20/F leg | Monophasic | 1 | + | +LS | + | + | – | + |
| 16 | PM* | 54/G arm | Monophasic | 2 | + | +LS | + | + | + | NE |
| 17 | PM* | 30/M elbow | Biphasic | 1 | + | +LS | + | + | + | + |
| 18 | PM* | 55/M shoulder | Monophasic | 1 | + | +LS | + | + | + | + |
| 19a | P | 16/F buttock | Monophasic | 2 | – | – | + | – | – | + |
| 19b | PM* | 18 | Monophasic | 2 | + | +L | NE | + | – | NE |
| 20a | P | 16/F thigh | Biphasic | 1 | + | +L | + | + | – | + |
| 20b | PM* | 19 | Biphasic | 1 | – | – | + | – | – | + |
| 20c | PM* | 20 | Biphasic | 1 | + | +LS | + | + | + | + |

ICC = immunocytochemical analysis; P = primary tumour; R = local tumour ; PM = pulmonary metastasis; AM = adrenal gland metastasis;
L = long form of SCF; S = short form of SCF; NE = no valuable; samples marked with an asterisk are from pre-treated patients.

© 2001 Cancer Research Campaign

SYT external 5′-CAACAGCAAGATGCATACCA-3′
SSX″ 5′-TGCTATGCACCTGATGACGA-3′

The annealing temperature was 52˚C for 1 min.

SYT internal 5′-AGACCAACACAGCCTGGACCA-3′
SSX4″ 5′-GGCACAGCTGTTTCCCATCA-3′

The annealing temperature was 58˚C for 1 min.

The amplification products of all the translocation breakpoints were analysed on 2% agarose gel in TAE1X buffer.

One of each fusion type transcript (SYT-SSX1, SYT-SSX2 and SYT-SSX4) was sequenced and used as positive control.

### c-Kit and SCF PCR

The c-Kit mRNA expression was evaluated using the primers (Ricotti et al, 1998)

5′ forward GAGTTGGCCCTAGAAGTTAGA
5′ reverse CCTGGAGGTGGATGCAAGTT

at the following conditions: 40 cycles at 94˚C for 1 min., 64˚C for 45 s, 72˚C for 1 min.

For its ligand, SCF, the amplification was carried out with the following primers

5′ forward ATTCAAGAGCCCAGAACCCA
5′ reverse CTGTTAACCAGCCAATGTACG

The PCR conditions were: 40 cycles at 94˚C for 1 min., 63˚C for 45 s, 72˚C for 1 min. The use of AmpliTaq Gold was recommended.

### c-Met and HGF PCR

c-Met receptor was amplified using the following primers (Moryama et al, 1995):

5′ forward ACAGTGGCATGTCAACATCGCT
5′ reverse GCTCGGTAGTCTACAGATTC

The used conditions were: 5 cycles 94˚C 1 min, 58˚C 2 min, 72˚C 2 min, 30 cycles 94˚C for 30 s, 59˚C for 1 min, 72˚C for 1 min. The ligand HGF, was amplified using the primers

5′ forward GGGAAATGAGAAATGCAGCCAG
5′ reverse AGTTGTATTGGTGGGTGCTTC

with the same conditions of c Met, using AmpliTaq Gold instead.

### Protein extraction

Proteins were extracted from tissue samples stored at –80˚C, by homogenization at 4˚C in lysis buffer (50 mM Tris-HCl pH 7.5, 5 mM EDTA, 150 mM NaCl, 0.5% Nonidet P-40) supplemented with protease inhibitors. Lysis was performed by frequent vortexing, followed by sonication step. Protein lysates were then cleared by centrifugation at 14 000 rpm at 4˚C for 30 min and quantitated through BCA analysis (Pierce, Rockford, USA).

### Immunoprecipitations and Western blotting

Equal amounts of lysates were subjected to immunoprecipitation by incubating with 3 µl of mouse monoclonal antibody Ab-3 (K45) (NeoMarkers, Union City, CA), directed against the c-Kit receptor, even if it was conjugated to its receptor (SCF). The M07e cell line lysate, with overexpressed c-Kit receptor, was used as positive control while the lysate from normal human synovia was used as the negative one. After a 2 h incubation with the specific antibody, at 4˚C and under gentle agitation, the protein complexes were incubated for 1 h with protein A Sepharose (Sigma, St. Louis, USA) at 4˚C with gentle agitation. Immunoprecipitates were then collected by centrifugation and washed three times with the same buffer supplemented with antiproteases. Next, the immunoprecipitated material was boiled for 5 min in Laemli buffer (62 mM Tris-HCl pH 6.8, 10% glycerol, 2% SDS, 0.003% bromophenole blue) and analysed by SDS-PAGE electrophoresis under reducing conditions on a 10% acrylamide minigel (Biorad, Richmond, CA). Coloured Rainbow protein markers (Amersham, UK) were used.

Proteins were transferred to a polyvinyldene difluoride membrane and subjected to Western blotting. The membrane was saturated with 4% BSA (Amersham) for 2 h at room temperature. c-Kit protein was detected with a rabbit polyclonal antibody (C-19, Santa Cruz Biotecnology, CA, USA) diluted 1:200 in TBS (20 mM Tris-HCl pH 7.5, 154 mM NaCl). The secondary antibody was used at the recommended dilution (Sigma). Specific bands corresponding to the investigated proteins were detected using a chemioluminescent technique, according to the manufacturer's recommendations (ECL detection system, Amersham).

Immunoprecipitation experiments against the β-actin protein were performed as previously described using 1 µl of the specific polyclonal antibody (Sigma) and using all the unbound total proteins derived form the c-Kit immunoprecipitation experiments. The subsequent Western blot was incubated with a mouse monoclonal β-Actin antibody (Sigma) diluted 1:5000.

### Immunocytochemical (ICC) analysis

ICC study was performed by the streptavidine-peroxidase conjugated method (Shi et al, 1998). The following primary antibodies were applied: anti c-Kit polyclonal rabbit antibody (C-19, Santa Cruz Biotechnology, Santa Cruz, CA, USA), anti c-Met polyclonal rabbit antibody (C-12, Santa Cruz Biotechnology, Santa Cruz, CA, USA), the monoclonal Bcl2 antibody (mouse clone 124, kindly donated by Dr Mason, Department of Histopathology, London University College) to paraffin-embedded sections using 1:100 and 1:50 dilutions, and 1:20 respectively.

The specificity of the primary antibodies c-Met and c-Kit were confirmed by the use of specific peptides (sc-168 P, Santa Cruz, for c-Kit and sc-10 P, Santa Cruz, for c-Met). Competition experiments were performed as follow: diluted antibody (1:50) was preincubated with 4 µg of the specific peptide and incubated at 37˚C for 2 h and then utilized at the working dilution following standard procedures.

Cytokeratin positivity was detected using a pool of antibody constituted by 34 βE12 (Dako) and KS 8.12 (Sigma) diluted 1:200, following standard procedures.

### RESULTS

Since, according to a recent report (Zhang et al, 1998) human mast cells can be a cellular source of SCF, and SS is known to bear in some cases a relevant number of this type of cell (Ueda et al, 1988), an evaluation of this cellular component has been performed. A relevant number of mast-cells was observed within the spindle cell component in some areas of surgical material sections in six cases (three biphasic and three monophasic) of SSs (case nos 3, 10, 13, 14, 16 and 20b of Table 1). However, none of the control sections of cryopreserved material showed a significant mast-cell component.

### SCF and c-Kit RNA expression

The mRNA of the c-Kit gene was detected in all the analysed samples but case nos 14, 19a and 20b of Table 1 (Figure 1A) and this expression profile parallels well with SCF mRNA expression. On a total of 20 positive cases, 14 tumours showed the specific SCF bands (494 bp and 409 bp, named L and S respectively) and 6 tumours (n. 5, 7, 8, 12, 19b and 20a) showed only the higher SCF band (L) (Figure 1B). The positive control M07e cell line displayed mRNA for both c-Kit receptor and its ligand represented by the two specific bands.

### c-Kit Immunoistochemical analysis

The immunostaining intensity was faint, diffuse and cytoplasmic in the spindle cells of monophasic SSs. In biphasic tumours a definite enhancement of reactivity was observed in the epithelial component which paralleled the switch off of the immunoreactivity of the spindle cells intermixed with or surrounded the epithelial nests/structures in most of the cases (Figures 2A and 2B).

Such an immunoreactivity was inhibited by the pre-incubation of the antibody with a specific synthetic peptide against which the antibody was raised.

Cytokeratin positivity, like c-Kit staining, was restricted to the epithelial cells (Figures 2E and 2F).

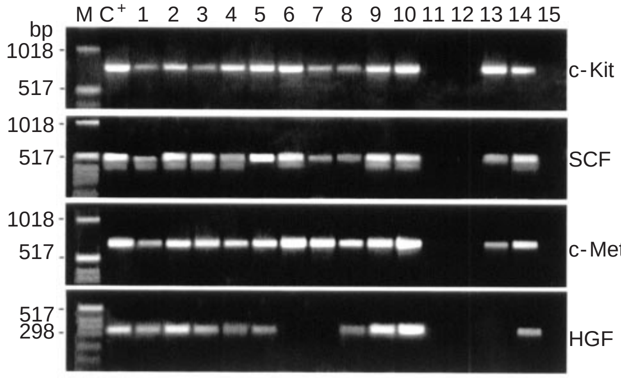

**Figure 1** RT-PCR analysis. RNA was reverse transcribed and the obtained cDNA was subjected to specific PCR. PCR products were run in a 1.8% agarose gel and photographed under UV light. Lane 1s to 14 correspond to case nos 1, 2, 3, 4, 5, 6, 7, 8, 11, 13, 14, 19a, 20a and 20c of Table 1. (**A**) mRNA expression of c-Kit. The detected band of 749 bp corresponds to c-Kit receptor. C+: as positive control mRNA from M07e cell line was used. (**B**) mRNA expression of SCF. Two bands of 494 and 409 bp corresponding to L-SCF and S-SCF respectively, were detected. C+: as positive control mRNA from M07e cell line was used. (**C**) mRNA expression of c-Met. A band of 656 bp corresponding to c-Met receptor was detected. C+: as positive control mRNA from FL-EBV was used. (**D**) mRNA expression of HGF. A band of 315–303 bp corresponding to HGF was detected. C+: as positive control mRNA from FL-EBV was used

### Immunoprecipitation of c-Kit receptor

Three cases where a large amount of frozen material was available, were chosen for immunoprecipitation experiments.

A band of 150 kDa, comigrating with the positive control of the c-Kit of M07e cell line, was detected in two biphasic synovial sarcomas (case nos 7 and 20a of Table 1) but not in one monophasic synovial sarcoma (case no. 11 of Table 1) and in the normal human synovia (Figure 3). As control, the bound proteic material derived form c-Kit IP was secondly immunoprecipitated with a specific antibody for β-Actin protein. The intensity of the detected band (50KDa) was identical in all the analysed cases (Figure 3). These results correlated to the ICC data even if case 11 showed both a c-Kit mRNA expression and reactivity with immunistochemical analysis.

### HGF and c-Met RNA expression

All cases were tested for the presence of HGF mRNA. Fourteen tumours showed the presence of the expected band and nine cases resulted negative (case nos 6, 7, 12, 14, 15, 19a, 19b, 20a and 20b of Table 1) (Figure 1D).

c-Met mRNA receptor was found in all tumours but cases 14, 19a and 20b (Figure 1C).

In six cases (6, 7, 12, 15, 19b and 20a) only the presence of c Met RNA, not associated to the presence of its ligand, HGF mRNA, was detected.

### c-Met immunoistochemical analysis

All the analysed tumours were positive but case nos 5 and 8 even if a mRNA expression was detected for both c-Met gene and its ligand. Two cases were not assessable cases (16 and 19b).

The areas overexpressing c-Met corresponded to the epithelial component of biphasic SSs, however a faint reactivity was present in the spindle cell component of both biphasic and monophasic tumours in accordance with previous reports (Kuhnen et al, 1998).

mRNA expression data (RT-PCR) (Table 1) did not show any correlation with pathologic stage (primary vs metastasis), histologic sub-type, fusion transcript and anatomical localization.

### DISCUSSION

In this study, 23 cases of primary and metastatic synovial sarcomas, 10 biphasic and 13 monophasic in sub-type, were analysed. We investigated the expression of c-Kit and its ligand, SCF, in addition to c-Met tyrosine kinase receptors and its ligand, HGF, by analysing the presence of related mRNAs. Western blot and immunocytochemical analysis were also performed thus confirming at protein level the expression of the two receptors.

To our knowledge, the present report is the first study that underlines the presence of the c-Kit/SCF system in SSs. Immunocytochemical analysis showed that the overexpression of this receptor is mainly restricted to the epithelial component of biphasic SSs. Accordingly, the band detected by immunoprecipitations and Western blot, corresponding to the c-Kit receptor, was only found in the proteic lysate obtained from two biphasic tumours and not in the monophasic one. The weak c-Kit expression observed in the monophasic subtype was probably insufficient to be detected by this technique. Unfortunately, due to the delayed snap-freezing of the tumour material, we were unable to assess the phosphorylation status of the receptor. Comparing the presence of

 © 2001 Cancer Research Campaign

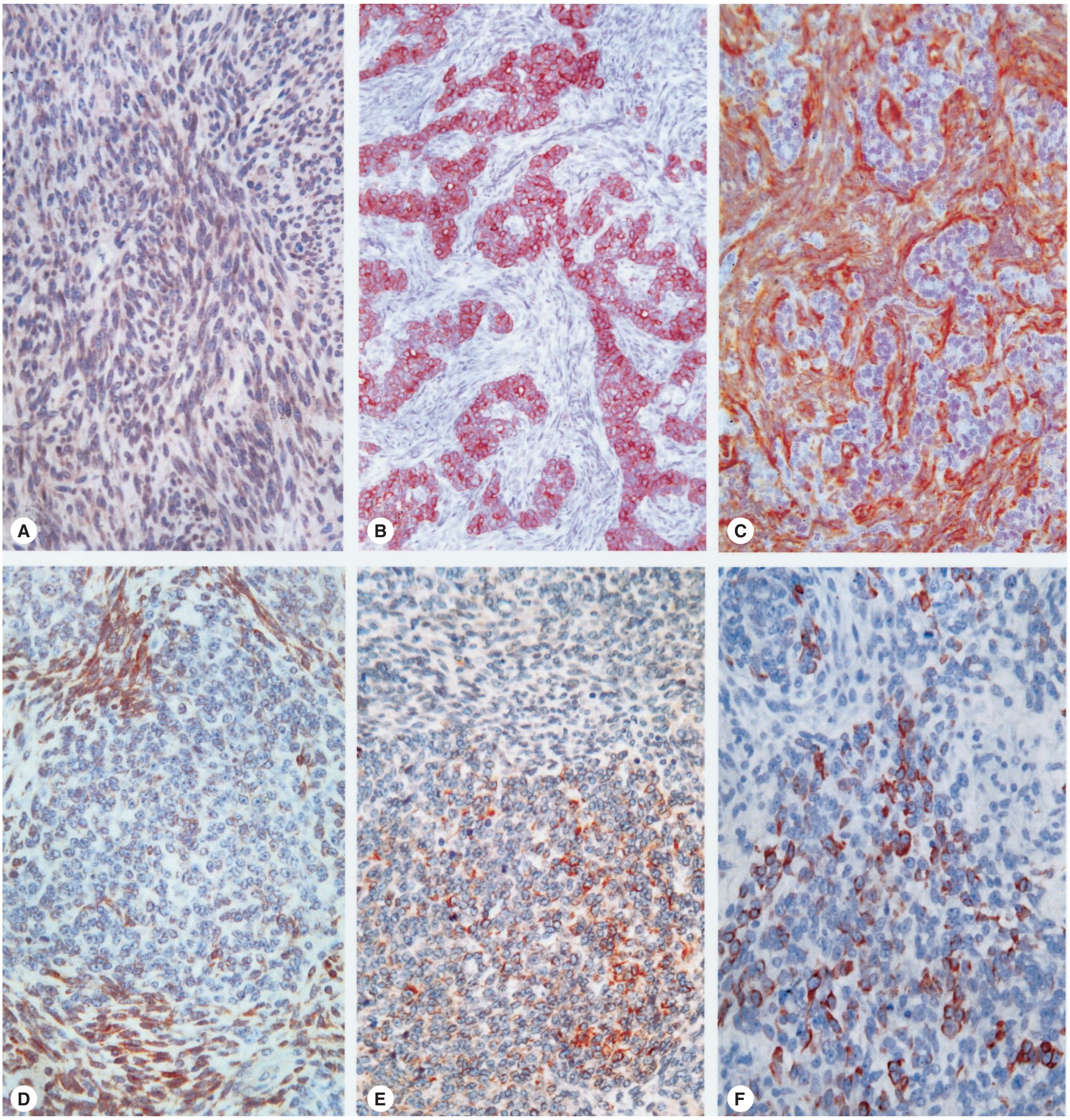

**Figure 2** ICC analysis. (**A**) Light expression of c-Kit in spindle cells of a monophasic SS (Table 1, case 6) (× 200). (**B**) Strong c-Kit cytoplasmic decoration restricted to epithelial component of a biphasic SS (Table 1, case 9) with evidence of switch off of c-Kit immunoreactivity in the surrounding spindle cells (× 200). (**C**) The same case showing bcl2 immunoreactivity reastricted to the spindle cell component (× 200). (**D**) Monophasic SS bcl2 stained (Table 1, case 5) (× 200). (**E**) Dot-like cytokeratin immunoreactivity which appears to parallel the weakness in bcl2 and the gain in c-Kit immunostaining in a monophasic SS with a plump cell component (× 200). (**F**) the same SS c-Kit immunodecorated (× 200)

both c-Kit and SCF mRNA we always observed a correlation between them: their tight association suggests therefore the existence of a possible autocrine loop in this type of tumour and we could thus hypothesize that the latter express a wild-type receptor. On the contrary, in the GISTs model, the constitutive phosporylation of the c-Kit receptor is due to point mutations in the exon 11 (Hirota et al, 1998), and makes redundant the production of the ligand, SCF, whose expression was always detected in our tumours.

In most of our cases, we identified two SCF isoforms, here defined L and S. The S isoform, lacking exon 6, encodes a membrane-bound SCF while isoform L encodes a protein containing a cleavage site that makes the ligand susceptible to post- translational proteolysis, yielding a soluble SCF. Both isoforms are biologically active (Anderson et al, 1990; Toskoz et al, 1992) but the mechanisms that control the tissue-specific and developmentally regulated production of SCF L vs SCF S are not well understood. In our SS series, we never detected the S isoform alone but we found the co-presence of the two isoforms L and S or the L form alone. The expression of c-Kit and of its ligand SCF, both as L or S form, did not show any segregation either to the

© 2001 Cancer Research Campaign 

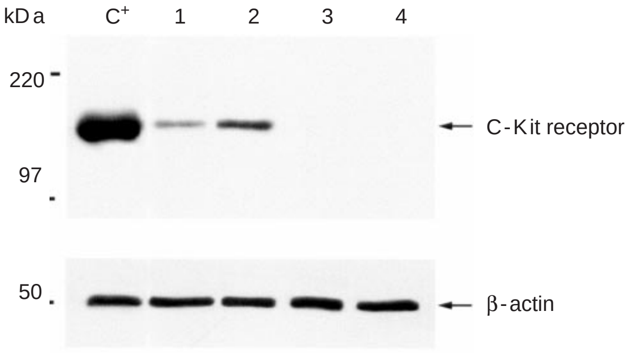

**Figure 3** Immunoprecipitation and Western blot analysis. Western blot with anti Kit immunoprecipitates from tumor specimens (lane 1 and 2: biphasic tumours cases n. 7 and 20a of Table 1; lane 3: monophasic tumour case n. 11 of Table 1) and lane 4: from normal tissue (synovia). All the samples were secondly immunoprecipitated with anti β-actin antibody. All lanes were reacted with ECL detection reagents according to manufacturer instructions pathologic tumour stage, histologic sub-type, fusion transcript, or anatomical localization. The possibility that the SCF mRNA transcripts in our cases were not derived from tumour cells but from contaminating mast cells (Ueda et al, 1988) was ruled out by the microscopic control on frozen sections of cryopreserved samples before molecular analysis.

The presence of c-Kit oncogene and its ligand, SCF, was recently reported in pPNETs (Ricotti et al, 1998). This finding is not unexpected since we know that undifferentiated SS may share some morphologic and immunophenotypic characteristics with pPNETs (Dei Tos et al, 1995; Folpe et al, 1998; van de Rijn et al, 1999) and that, though rarely, SSs may show pPNET featuring components (Mezzelani et al, 1998; Noguera et al, 1998).

A concomitant mRNA expression of HGF and c-Met genes was firstly reported in cell lines derived from other tumours of mesenchymal origin, such as a human glioma (Moryama et al, 1995) and human osteosarcoma cell lines (Cooper et al, 1984). More recently, c-Met protein expression was reported in two different series of SSs (Motoi et al, 1998; Kuhnen et al, 1998). In order to complement our c-Kit data, we investigated the expression of c-Met and its ligand, HGF, along with c-Met protein. Although our molecular results did not completely fit in with the immunocytochemical ones and some discrepancies need further investigation, immunophenotyping results were superimposable to those already described by Kuhnen and colleagues. We detected in fact, a strong expression of c-Met gene product in the epithelial component and a faint reactivity in the spindle cell one. Since it is currently conceived that each SS epithelial and spindle tumour cell harbours the same type of translocation t(X;18) (Clark et al, 1994), the evidence that the two TK related genes, c-Met and c-Kit, even though more expressed in the biphasic SS are also expressed in monophasic subtype, gives further support to the hypothesis that the two SS subtypes may truly represent a ‘carcinosarcoma’ with differently developed epithelial component (Leader et al, 1987; Miettinen et al, 1984).

Cumulatively, the current evidence suggests that the non- random SS translocation t(X;18) may trigger the transactivation of a cascade of genes such as c-Met, c-Kit, and their ligands, yielding two different autocrine loops: the first acting on the epithelial differentiation and the second working in a program protecting from apoptosis (Timeus et al, 1997). Since in a previous study (Mancuso et al, 2000) we have shown that a Bcl2 over-expression is a part of the SS gene profile and this expression, at variance with c-Kit receptor, is mainly restricted to the spindle cell component, the scenario that can be envisaged might be as follows. The epithelial differentiation process is driven by the c-Met gene and this driving parallels the shifting of the antiapoptotic role from Bcl2 to the c-Kit gene during transition from the spindle uncommitted to the epithelial differentiated cells. The shifting is in keeping with the tight correlation between the cell type and both modulation of expression and specific localization of the c-Kit gene product we observed. In fact, in biphasic SS the faint decoration of the spindle cells tends to disappear in the spindle, albeit Bcl2 positive, cells, surrounding the epithelial areas to which the strongest c-Kit immunoreactivity is restricted (Figure 2A–C) and the cytokeratin positivity confirm this shifting (Figure 2E). Moreover, primary biphasic tumours may reoccur as monophasic SSs (Mancuso et al, 2000). Again this does not appear a contradictory finding since the modulations and changes of morphologic features that occur and that we observed during recurrences or metastatic progression, may be ascribed to the dynamic plasticity of the expression of the involved genes.

### ACKNOWLEDGEMENTS

This work was supported by AIRC (Associazione Italiana per la Ricerca sul Cancro), grant n.420.198.122 and by “Ministero della Sanita” Ricerca Finalizzata 1998-ICSO30. 1/RF98.32-Italy. The authors thank Mrs Marina Sperni for reviewing the English format of the manuscript and Mr Mario Azzini for photographic assistance.

### REFERENCES

Adams V, Kempf W, Hassam S and Briner J (1995) Determination of Hexokinase isoenzyme I and II composition by RT-PCR: increased hexokinase isoenzyme II in human renal cell carcinoma. *Biochem Mol Med* **54**: 53–58

Anderson DM, Lyman SD, Baird A, Wignall JM, Eisenman J, Rauch C, March CJ, Boswell HS, Gimpel SD, Cosman D and Wiliams DE (1990) Molecular cloning of mast cell growth factor, a hematopoietin that is active in both membrane bound and soluble forms. *Cell* **63**: 235

Besmer P, Murphy JE, George PC, Qui F, Bergold PJ, Lederman L, Snyder Jr. HW, Brodeur D, Zuckerman EE and Hardy WD (1986) A new acute transforming feline retrovirus and relationship of its oncogene v-kit with the protein kinase gene family. *Nature* **320**: 415–421

Brizzi MF, Zini MG, Aronica MG, Blechman JM, Yarden Y and Pegoraro L (1994) Convergence of Signaling by Interleukin-3, granulocyte-macrophage-colony-stimulating factor, and mast cell growth factor on JAK2 Tyrosine Kinase. *JBC* **269**(50): 31680–31684

Clark J, Rocques PJ, Crew AJ, Gill S, Shipley J, Chan AM, Gusterson BA and Cooper CS (1994) Identification of novel genes, SYT and SSX, involved in the t(X;18)(p11.2;q11.2) translocation found in human synovial sarcoma. *Nat Genet* **7**: 502–508

Cooper CS, Park M, Blair D, Tainsky MA, Heubner K, Croce CM and Vande Woude GF (1984) Molecular cloning of a new transforming gene from a chemically transformed human cell line. *Nature* **311**: 29–33

Copeland NG, Gilbert DJ, Cho BC, Donovan PJ, Jenkins NA, Cosman D, Anderson D, Lyman SD, and Williams DE (1990) Mast cell growth factor maps near the steel locus on mouse chromosome 10 and is deleted in a number of steel alleles. *Cell* **63**: 175–183

Dei Tos AP, Wadden C, Calonie E, Sciot R, Pauwels P, Knight JC, Dal Cin P and Fletcher CDM (1995) Immunohistochemical demonstration of glycoprotein p30/32 MIC2 (CD99) in synovial sarcoma. A potential cause of diagnostic confusion. *Applied Immunohist* **3**(3): 168–174

Folpe AW, Schmid RA, Champman D and Gown AM (1998) Poorly differentiated Synovial sarcoma. Immunoistochemical distinction from primitive neuroectodermal tumors and high grade malignant peripheral nerve sheath tumors. *Am J Sur Pathol* **22**(6): 673–680

 *© 2001 Cancer Research Campaign*

Fukuda T, Ichimura E, Shinozaki T, Sano T, Kashiwabara K, Oyama T, Nakajiama T and Nakamura T (1998) Coexpression of HGF and c-Met receptor in human bone and soft tissue tumors. *Path Int* **48**: 757–762

Galli SJ, Tsai M and Wershil BK (1993) The c-Kit receptor, stem cell factor and mast cells. What each is teaching us about the others. *Am J Pathol* **142**: 965–974

Hirota S, Isozaki K, Moriyama Y, Hashimoto K, Nishida T, Ishiguro S, Kawano K, Hanada M, Kurata A, Takeda M, Tunio GM, Matsuzawa Y, Kanakura H, Shinomura Y and Kiramura Y (1998) Gain-of-function of c-Kit in human gastrointestinal stromal tumors. *Science* **279**: 577–580

Hirota S, Okazaki T, Kitamura Y, O'Brien P, Kapusta L and Dardick I (2000) Cause of familial and multiple gastrointestinal autonomic nerve tumors with hyperplasia of interstitial cells of Cajal is germeline mutation of the c-Kit gene. *Am J Surg Pathol* **24**(2): 326–327

Kawai A, Woodruff J, Healey JH, Brennan MF, Antonescu CR and Ladanyi M (1998) SYT-SSX gene fusion as a determinant of morphology and prognosis in synovial sarcoma. *N Engl J Med* **338**: 153–160

Kindblom LG, Remotti HE, Aldenborg F and Meis-Kindblom JM (1998) Gastrointestinal pacemaker cell tumor (GIPACT). Gastrointestinal strom tumors show phenotypic characteristics of the interstitial cells of Cajal. *Am J Pathol* **152**: 1259–1269

Kuhnen C, Tolnay E, Steinau HU, Voss B and Muller KM (1998) Expression of c-met receptor and hepatocyte growth factor/scatter factor in Synovial sarcoma and epithelioid sarcoma. *Virch Arch* **432**: 337–342

Lasota J, Jasinski M, Darlomo-Rikala M and Miettinen M (1999) Mutation in exon 11 of c-Kit occur preferentially in malignant versus benign gastrointestinal stromal tumors and do not occur in leiomyomas or leiomyosarcomas. *Am J Pathol* **154**: 53–60

Leader M, Patel J, Collins M and Kristin H (1987) Synovial sarcomas. True carcinosarcomas? *Cancer* **59**: 2096–2098

Mancuso T, Mezzelani A, Riva C, Fabbri A, Dal Bo L, Sampietro G, Perego P, Casali P, Zunino F, Sozzi G, Pierotti MA and Pilotti S (2000) Analysis of SYT-SSX fusion trascript and bcl2 expression and phosphorylation status in synovial sarcoma. (In press) *Lab Invest* 80: 805–813

Mezzelani A, Sozzi G, Pierotti MA and Pilotti S (1998) Morpho-Immunophenotypic-Genotypic infidelity in synovial sarcoma. *Diagn Mol Pathol* **7**: 232–233

Miettinen M and Virtanen I (1984) Synovial Sarcoma: a misnomer. *Am J Pathol* **117**: 18–25

Moryama T, Kataoka H, Tsubouchi H and Koono M (1995) Concomitant expression of hepatocyte growth factor (HGF), HGF activator and c-met genes in human glioma cells in vitro. *FEBS* **372**: 78–82

Moskaluk CA, Tian Q, Marshall CR, Rumpel CA, Franquemont DW and Frierson HF (1999) Mutations of c-Kit JM domain are found in a minority of human gastrointestinal stromal tumors. *Oncogene* **18**: 1897–1902

Motoi T, Ishida T, Kuroda M, Horiuchi H, Oka T, Matsumoto K, Nakamura T and Machinami R (1998) Co-expression of hepatocyte growth factor and c-Met proto-oncogene product in Synovial Sarcoma. *Path Int* **48**: 769–775

Nishida T, Hirota S, Taniguchi M, Hashimoto K, Isozaki K, Nakamura H, Kanakura H, Tanaka T, Takabayashi A, Matsuda H and Kiramura Y (1998) Familial gastrointestinal stromal tumors with germline mutations of the c-Kit gene. *Nature Genetics* **19**: 323–324

Nocka K, Buck J, Levi E and Besmer P (1990) Candidate ligand for the c-Kit transmembrane kinase receptor. KL, a fibroblast derived growth factor stimulates mast cells and erythroid progenitors. *EMBO J* **9**: 3287–3294

Noguera R, Navarro S, Cremades A, Rosello'-Sastre E, Pellin A, Peydro'-Olaya A and Llombardt-Bosch A (1998) Translocation (X;18) in a biphasic Synovial Sarcoma with morphologic features of neural differentiation. *Diag Mol Patol* **7**(1): 16–23

Oda Y, Sakamoto A, Saito T, Kinukawa N, Iwamoto Y and Tsuneyoshi M (2000) Expression of Hepathocyte Growth factor (HGF)/scatter factor and its receptor c-Met correlates with poor prognosis in Synovial Sarcoma. *Hum Pathol* **31**(2): 185–192

Qui FH, Ray P and Brown K (1987) Primary structure of c-Kit: relationship with the CSF-1/PDGF receptor kinase family-oncogenic activation v-kit involves deletion of extracellular domain and C terminus. *EMBO J* **7**: 1003–1011

Ricotti E, Fagioli F, Garelli E, Linari C, Crescenzio N, Horenstein AL, Pistamiglio P, Vai S, Berger M, Cordero di Montezemolo L, Madon E and Basso G (1998) c-Kit is expressed in soft tissue sarcoma of neuroectodermic origin and its ligand prevents apoptosis of neoplastic cells. *Blood* **91**: 2397–2405

Shi ZR, Itzkowitz SH and Kim YS (1988) A comparison of three immunoperoxidase techniques for antigen detection in colorectal carcinoma tissues. *J Histochem Cytochem* **36**: 317–322

Timeus F, Crescenzio N, Valle P, Pistamiglio P, Piglione M, Garelli E, Ricotti E, Rocchi P, Strippoli PL, Cordero di Montezemolo L, Madon E, Ramenghi U and Basso G (1997) Stem cell factor suppresses apoptosis in neuroblastoma cell line. *Exp Hematol* **25**: 1253–1260

Torihashi S, Nishi K, Tokutomi Y, Nishi T, Ward S and Sanders KM (1999) Blockade of kit signaling induces transdifferentiation of interstitial cells of Cajal to a smooth muscle phenotype. *Gastroenterology* **117**(1): 140–148

Toskoz D, Zsebo KM, Smith KA, Brankow D, Suggs SV, Martin FH and Williams DA (1992) Support of human hematopoiesis in long term bone marrow cultures by murine stromal cells selectively expressing the membrane bound and secreted forms of the human homolog of the steel gene product, stem cell factor. *PNAS* **89**: 7350

Ueda T, Aozasa K, Tsujimoto M, Yoshikawa H, Kato T, Ono K and Matsumoto K (1988) Prognostic significance of Mast cells in soft tissue sarcoma. *Cancer* **62**: 2416–2419

van de Rijn M, Barr FG, Xiong QB, Hedges M, Shipley J and Fisher C (1999) Poorly differentiated Synovial sarcoma. An analysis of clinical, pathologic and molecular genetic features. *Am J Sur Pathol* **23**(1): 106–112

Williams DE, Eisenman J, Baird A, Rauch C, Ness VK, March CJ, Park LS, Martin U, Mochizuki DY and Boswell HS (1990) Identification of a ligand for the c-Kit proto-oncogene. *Cell* **63**: 167–174

Xie Y, Skytting B, Nilsson G, Brodin B and Larsson O (1999) Expression of insulin like growth factor 1 receptor in Synovial Sarcoma: association with an aggressive phenotype. *Cancer Res* **59**: 3588–3591

Yarden Y, Kuang WJ and Yang-Feng T (1987) Human proto-oncogene c-Kit: a new cell surface receptor tyrosine kinase for an unidentified ligand. *EMBO J* **6**: 3341–3351

Zhang S, Anderson DF, Bradding P, Coward WR, Baddeley SM, MacLeod JDA, McJill JI, Church MK, Holgate ST and Roche WR (1998) Human mast cells express stem cell factor. *J Pathol* **186**: 59–66

*© 2001 Cancer Research Campaign*
